# Additive methods for genomic signatures

**DOI:** 10.1186/s12859-016-1157-8

**Published:** 2016-08-22

**Authors:** Rallis Karamichalis, Lila Kari, Stavros Konstantinidis, Steffen Kopecki, Stephen Solis-Reyes

**Affiliations:** 1 School of Computing Science, University of Waterloo, Waterloo, ON, N2L 3G1, Canada; 2 Department of Computer Science, University of Western Ontario, London ON, N6A 5B7, Canada; 3 Department of Mathematics and Computing Science, Saint Mary’s University, Halifax NS, Canada

**Keywords:** Comparative genomics, Alignment-free, Genomic signature, Chaos Game Representation, Information distance, Additive DNA signature, Composite DNA signature, Assembled DNA signature

## Abstract

**Background:**

Studies exploring the potential of Chaos Game Representations (CGR) of genomic sequences to act as “genomic signatures” (to be species- and genome-specific) showed that CGR patterns of nuclear and organellar DNA sequences of the same organism can be very different. While the hypothesis that CGRs of mitochondrial DNA sequences can act as genomic signatures was validated for a snapshot of all sequenced mitochondrial genomes available in the NCBI GenBank sequence database, to our knowledge no such extensive analysis of CGRs of nuclear DNA sequences exists to date.

**Results:**

We analyzed an extensive dataset, totalling 1.45 gigabase pairs, of nuclear/nucleoid genomic sequences (nDNA) from 42 different organisms, spanning all major kingdoms of life. Our computational experiments indicate that CGR signatures of nDNA of two different origins cannot always be differentiated, especially if they originate from closely-related species such as *H. sapiens* and *P. troglodytes* or *E. coli* and *E. fergusonii*. To address this issue, we propose the general concept of additive DNA signature of a set (collection) of DNA sequences. One particular instance, the composite DNA signature, combines information from nDNA fragments and organellar (mitochondrial, chloroplast, or plasmid) genomes. We demonstrate that, in this dataset, composite DNA signatures originating from two different organisms can be differentiated in all cases, including those where the use of CGR signatures of nDNA failed or was inconclusive. Another instance, the assembled DNA signature, combines information from many short DNA subfragments (e.g., 100 basepairs) of a given DNA fragment, to produce its signature. We show that an assembled DNA signature has the same distinguishing power as a conventionally computed CGR signature, while using shorter contiguous sequences and potentially less sequence information.

**Conclusions:**

Our results suggest that, while CGR signatures of nDNA cannot always play the role of genomic signatures, composite and assembled DNA signatures (separately or in combination) could potentially be used instead. Such additive signatures could be used, e.g., with raw unassembled next-generation sequencing (NGS) read data, when high-quality sequencing data is not available, or to complement information obtained by other methods of species identification or classification.

**Electronic supplementary material:**

The online version of this article (doi:10.1186/s12859-016-1157-8) contains supplementary material, which is available to authorized users.

## Background

Motivated by the general need to identify and classify species based on molecular evidence, alignment-free genome comparisons have been proposed, based on comparing Chaos Game Representations (CGR) of genomic DNA sequences. The CGR of a DNA sequence, proposed by Jeffrey [1, 2], is a graphical representation of a DNA sequence, where the patterns in the image correspond to the frequencies of *k*-mers in the sequence. Deschavanne et al. [3, 4] were the first to suggest that CGR is a good candidate for the role of “genomic signature” defined by Karlin and Burge [5] as any specific quantitative characteristic of a sequence that is pervasive along the genome, while being dissimilar for sequences originating from organisms of different species.

CGR is one of a variety of alignment-free methods (see [6–11] for detailed literature reviews) that have been proposed for sequence and genome comparisons, as a computationally efficient approach that performs well even with DNA sequences that have nothing or little in common. (We use the following notational conventions for genomic DNA: nDNA (nuclear/nucleoid DNA), mtDNA (mitochondrial DNA), cpDNA (chloroplast DNA), and pDNA (plasmid DNA)).

Initially, CGR images were only qualitatively analyzed [12–14], and Dutta et al. and Goldman both advanced the suggestion that CGR images represent no more information than second-order Markov chains [15, 16], which was later disproven by Almeida et al. [17, 18] and others [19, 20]. CGR has been applied extensively to phylogenetics together with the Euclidean distance, for instance on nDNA fragments from various domains [3], 27 genomes from various genera [4], 125 nDNA fragments from several bird genomes [21], 26 mtDNA sequences (also with the Pearson distance and a custom image distance) [19], 4 bacteria and about 200 phages [22], 75 HIV-1 genomes [23], 10 mtDNA sequences and 14 nDNA sequences from plants in the *Brassicales* order [24]. Other distances have also been used, for instance the DSSIM image distance on a set of 3,176 mtDNA sequences [20], and six different distances on 174 million base pairs of sampled nDNA fragments from organisms of all major kingdoms of life [25]. The performance of several distance functions has also been compared and benchmarked on their accuracy in constructing phylogenetic trees [26–32]. Initially, CGR was used only for strings over a 4-letter alphabet (like DNA), but generalizations have been proposed to peptide sequences [33–38], and Almeida and Vinga proposed a derivative of CGR called the Universal Sequence Map (USM), which is suitable for alphabets of any size [39, 40]. CGRs have also been subjected to multifractal analysis (which measures the degree of self-similarity within the image), see, e.g., [35, 41–46]. Lastly, CGR has been used to estimate sequence entropy [47–49], to speed up local-alignment algorithms [50], and has been used together with neural networks to classify HPV genomes by genotype [51].

Several CGR studies [13, 20, 52] observed that CGR patterns of nuclear and organellar DNA sequences of the same organism can be completely different. While the hypothesis that CGRs of mitochondrial DNA sequences can play the role of genomic signatures was tested and validated on the set of all 3,176 sequenced mitochondrial genomes (totalling 91.3 megabase pairs) available in the NCBI GenBank sequence database in July 2012 [20], to our knowledge no such extensive analysis of CGRs of nuclear/nucleoid genomic sequences exists to date.

The main contributions of this paper are:

- We present an extensive analysis of the hypothesis that conventionally computed (called herein “conventional”) nDNA signatures can play the role of genomic signatures at multiple taxonomic levels, from kingdom to species. Our dataset totals 1.45 gigabase pairs of nDNA sequences from 42 different genomes, from all major kingdoms of life.
- Our analysis indicates that conventional nDNA signatures of two different origins cannot always be differentiated, especially if they originate from closely related organisms. To address this issue, we propose taking into account information obtained from organellar DNA, in addition to nDNA. More generally, we propose the concept of an additive DNA signature of a set (collection) of DNA sequences, and define two particular instances: composite DNA signatures and assembled DNA signatures.
- We explore composite DNA signatures, which combine conventional nDNA signatures with organellar DNA signatures (mtDNA, cpDNA, or pDNA) of the same organism. We demonstrate that, in this dataset, the composite DNA signatures originating from two different organisms can be differentiated in all cases, including those where the use of conventional nDNA signatures failed. In particular, composite DNA signatures from genomes of species as closely related as *H. sapiens* and *P. troglodytes*, or *E. coli* and *E. fergusonii*, can be successfully separated.
- We explore assembled DNA signatures, which combine information from many short contigs (e.g., 100 bp) of a DNA fragment to produce a recognizable signature. This is in contrast to conventional DNA signatures wherein one single long (thousand to hundreds of thousands of basepairs) DNA sequence is needed to generate a recognizable signature.

The enhanced discriminating power of composite DNA signatures, and the ability of assembled DNA signatures to operate with scattered and reduced sequence data, open the possibility of practical applications including aiding species identification or classification, and comparisons of DNA fragments of various origins such as genomes of extinct organisms, synthetic genomes, raw unassembled next-generation sequencing (NGS) read data, or even computer-generated DNA sequences.

## Results

The first objective of this study was to test, on a comprehensive dataset, the hypothesis that conventional nDNA signatures can be used to differentiate between nuclear DNA sequences originating from different organisms, spanning all major kingdoms of life, at multiple taxonomic levels.

To this end, the following computational experiment was performed, for each of the major kingdoms of life, at various taxonomic levels. We chose a pivot organism (e.g., *H. sapiens* for Kingdom Animalia) and proceeded to use conventional nDNA signatures to compare fragments of its nuclear/nucleoid genome with fragments of the nuclear/nucleoid genome of one other organism from the same kingdom. The process was then repeated with the second organism being at increasing degrees of relatedness to the pivot organism.

More precisely, for each such pairwise comparison, the following three-step process was implemented.

- Randomly sample 150 kbp nDNA fragments from every chromosome (20 per chromosome, or all fragments if fewer) of the two genomes involved in the comparison. For each such nDNA fragment, construct its corresponding conventional nDNA signature using the process described in Section “Methods”.
- Compute pairwise distances for all pairs of conventional nDNA signatures generated in Step 1. The distance used to start with was an approximated information distance (AID), formally defined in Section “Methods” (see also [25, 53]), since it is computationally simple and uses the least amount of sequence information. If separation was not achieved using AID, five other distance measures were used: Structural Dissimilarity Index (DSSIM) [54], Euclidean distance, Pearson correlation distance [55], Manhattan distance [56], and descriptor distance [25].
- Use the distance matrix obtained in Step 2 as input to a Multi-Dimensional Scaling (MDS) algorithm to produce a 3D Molecular Distance Map [25]: Each point in the map corresponds to (the conventional nDNA signature of) an nDNA fragment from Step 1, and the geometric distance between every two points corresponds to the distance between the respective conventional nDNA signatures in the distance matrix. Assess, for each Molecular Distance Map, whether or not separation between conventional nDNA signatures of DNA fragments from the pivot organism and those from the other organism was achieved, by using either *k*-means clustering [57] or by verifying the existence of a separating plane.

**Fig. 1:**
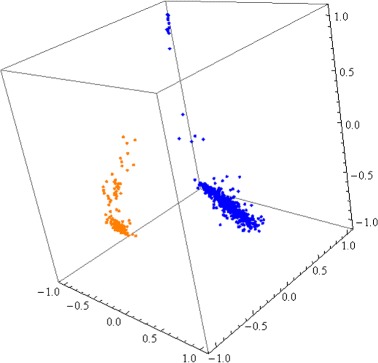
3D Molecular Distance Map illustrating interrelationships among conventional nDNA signatures of 480 randomly sampled 150 kbp nuclear genomic fragments from *H. sapiens* (*blue*) and 128 randomly sampled 150 kbp nuclear genomic fragments from *D. melanogaster* (*orange*). The accuracy of separation is 97.2 %

Figure 1 illustrates an example of the end result of this three-step process: A three-dimensional Molecular Distance Map that displays the conventional nDNA signatures of the pivot organism of Kingdom Animalia, *H. sapiens*, plotted together with the conventional nDNA signatures of *D. melanogaster*.

**Fig. 2:**
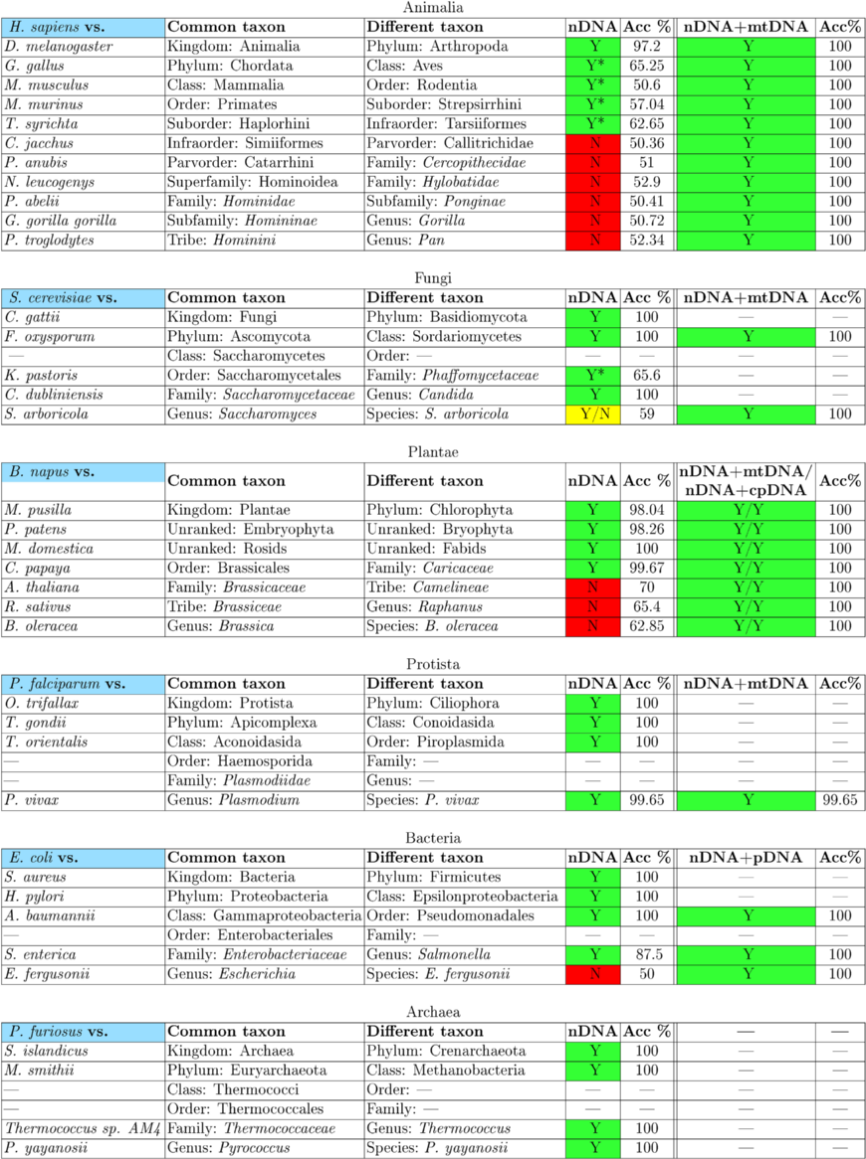
Each subfigure summarizes, for a given kingdom, the results of pairwise comparisons between DNA signatures of fragments from a pivot organism (*blue*) and those from one other organism, at increasing levels of relatedness. The first two result columns indicate the outcome of the comparisons of conventional nDNA signatures, and the last two columns the comparisons of composite DNA signatures. *Green* indicates that separation was achieved with AID, *red* indicates that separation was not achieved with any of the six distances listed in Section “Results”, and yellow (Y/N) or Y* indicate results discussed in the text. The columns labelled Acc % indicate the accuracy of the separations listed immediately at their left: *A* *c* *c*>85 *%* was considered separation. A dash indicates that no sequenced data was available on NCBI/GenBank at the time of this submission. The corresponding 3D Molecular Distance Maps for each of the comparisons can be found in [58]

The results for all kingdoms are presented in Fig. 2 (the first two result columns) and the corresponding 3D Molecular Distance Maps can be found in [58]. For Kingdom Animalia, the approximated information distance succeeded to separate *H. sapiens* (24 chromosomes, 480 fragments) conventional nDNA signatures from those of other organisms, down to and including from *M. murinus* (grey mouse lemur, same order but different suborder) and *T. syrichta* (Phillipine tarsier, same suborder but different infraorder). In the cases marked Y* in Fig. 2, while the accuracy was less than the threshold for separation (85 *%*), the existence of a separating plane was verified. See discussion in Section “Methods” for details.

**Fig. 3:**
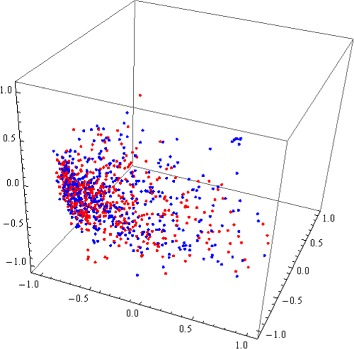
3D Molecular Distance Map illustrating interrelationships among conventional nDNA signatures of 480 randomly sampled nuclear genomic fragments from *H. sapiens* (*blue*) and 500 randomly sampled nuclear genomic fragments from *P. troglodytes* (*red*). All fragments are 150 kbp long, the accuracy of separation is 52.34 %, and no separation plane could be found

The use of conventional nDNA signatures failed to achieve separation for genomes of more closely related species. In particular, it failed to separate conventional nDNA signatures of *H. sapiens* from those of *C. jacchus* (common marmoset, same infraorder), *P. anubis* (Anubis baboon, same parvorder), *N. leucogenys* (northern white-cheeked gibbon, same superfamily), *P. abelii* (Sumatran orangutan, same family), *G. gorilla* (gorilla, same subfamily, and *P. troglodytes* (chimpanzee, same tribe, see Fig. 3). For those organisms where separation was not achieved with approximated information distance, we performed the comparisons with the other five distances. The results of these multiple computations were that, in all cases where approximated information distance failed to achieve separation, the other distances also failed.

For Kingdom Fungi, the pivot organism is the model organism *Saccharomyces cerevisiae* (16 chromosomes, 73 fragments), a species of yeast instrumental to winemaking, baking, and brewing. Separation of its conventional nDNA signatures was achieved down to and including separation from *C. dubliniensis* (same family, different genus). In the case of the comparison with *K. pastoris*, marked with Y* in Fig. 2, the accuracy score was lower than 85 *%*: This is an artifact of the shape of the 3D Molecular Distance Map wherein one of the clusters has a trailing set of points that become erroneously separated by *k*-means from all the rest of the points. Because of this, and since the use of *k*-means on the 2D Molecular Distance Map of the same dataset resulted in an accuracy score of 100 %, we interpreted this comparison as resulting in separation. The results of the comparison between the conventional nDNA signatures of the pivot organism and those of *S. arboricola* (same genus, different species), were inconclusive: The use of Euclidean and Pearson distances resulted in separation (both with accuracy of 88.48 %), while the use of the other four distances (DSSIM, Manhattan, descriptor, approximated information distance) did not result in separation.

For Kingdom Plantae, the pivot organism is the model organism *Brassica napus* (19 chromosomes, 380 DNA fragments), rapeseed, a flowering member of the family *Brassicaceae* (mustard or cabbage family). Separation of its conventional nDNA signatures was achieved down to and including separation from *C. papaya* (papaya, same order, different family). For the comparisons with *A. thaliana* (thale cress, same family, different tribe) and *R. sativus* (radish, same tribe, different genus), cluster separation was visually observed but not quantitatively confirmed by either *k*-means or plane separation. The comparison with *B. oleracea* (wild cabbage, same genus, different species) did not result in separation, with any of the six distances.

For Kingdom Protista, the pivot organism is the model organism *Plasmodium falciparum*, a protozoan parasite (14 chromosomes, 149 DNA fragments), one of the species of *Plasmodium* that cause malaria in humans. Separation of its conventional nDNA signatures from those of other organisms from the same kingdom was achieved at all taxonomic levels, down to and including separation from *P. vivax* (same genus, different species).

For Kingdom Bacteria, the pivot organism is the model organism *Escherichia coli* (20 genomic DNA fragments), a bacterium commonly found in the lower intestine of warm-blooded organisms. Separation of its conventional nDNA signatures from those of other bacteria was successful down to and including separation from *S. enterica* (same family, different genus), but failed with all six distances in the comparison with *E. fergusonii* (same genus, different species).

For Kingdom Archaea, the pivot organism is the model organism *Pyrococcus furiosus* (12 genomic DNA fragments), an extremophilic species of Archaea. Separation of its conventional nDNA signatures from those of other archaea was successful at all levels, down to and including separation from *P. yayanosii* (same genus, different species).

The above results indicate that, especially in Kingdom Animalia, conventional nDNA signatures cannot always be used to differentiate nuclear/nucleoid genomic sequences originating from two different genomes. This suggests that conventional nDNA signatures cannot always play the role of a “genomic signature”, particularly when the genomes being compared belong to closely related species.

### Composite DNA signatures

To enhance the discriminating power of conventional nDNA signatures, our second objective was to introduce and explore the concept of composite DNA signatures, which combine conventional nuclear/nucleoid DNA signatures with signatures of organellar genomes (mtDNA, cpDNA, or pDNA).

To test the discriminating power of composite DNA signatures, we repeated all previous pairwise comparisons (where sequenced organellar DNA was available), using this time composite DNA signatures. The results are presented in the last two columns of Fig. 2.

**Fig. 4:**
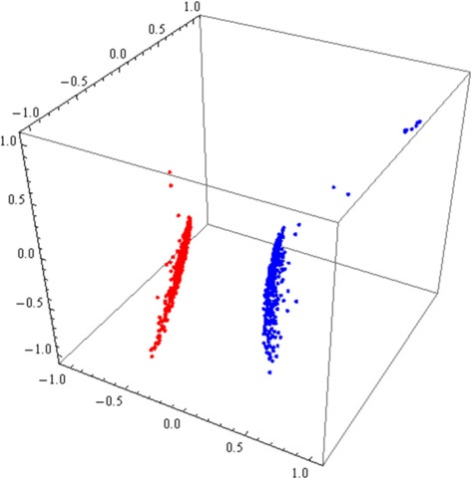
3D Molecular Distance Map illustrating interrelationships among composite DNA signatures using nDNA and mtDNA, of 480 DNA fragments from *H. sapiens* (*blue*) and 500 DNA fragments from *P. troglodytes* (*red*). The accuracy of separation is 100 %

For Kingdoms Animalia, Fungi and Protista we used composite DNA signatures combining the conventional nDNA signature of each nuclear/nucleoid genomic fragment with that of the mtDNA of the same organism (when available). Using such composite DNA signatures, differentiation of DNA signatures by organism was successful in all cases, including all cases where the use of conventional nDNA signature previously failed or was inconclusive. See Fig. 3 (*H. sapiens* vs. *P. troglodytes* conventional nDNA signatures, no separation) versus Fig. 4 (*H. sapiens* vs. *P. troglodytes* composite DNA signatures using nDNA and mtDNA, complete separation).

**Fig. 5:**
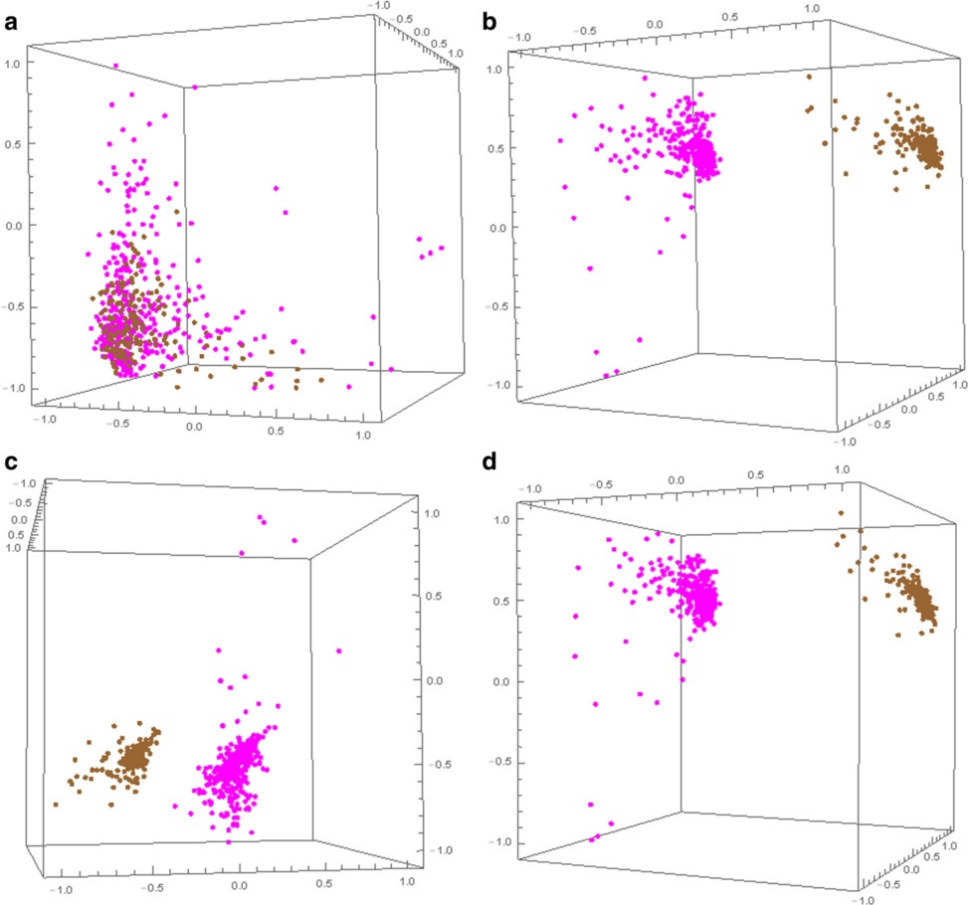
3D Molecular Distance Map illustrating interrelationships among signatures of 380 DNA fragments from *B. napus* (*magenta*) and 180 DNA fragments from *B. oleracea* (*brown*) using **a** conventional nDNA signatures, **b** composite DNA signatures using nDNA and mtDNA, **c** composite DNA signatures using nDNA and cpDNA, and **d** composite DNA signatures using nDNA, mtDNA, and cpDNA. The accuracy of separation is 63.03 % for (**a**), and 100 % for each of (**b**), (**c**), and (**d**)

To test the discriminating power of composite DNA signatures using nDNA, mtDNA and cpDNA, we employed them to perform comparisons for all genome pairs from Kingdom Plantae. Separation was achieved using all of: composite DNA signatures using nDNA and mtDNA, composite DNA signatures using nDNA and cpDNA, and composite DNA signatures using nDNA, mtDNA, and cpDNA. See Fig. 5 for the Molecular Distance Maps illustrating the relationships between these signatures for *B. napus* and *B. oleracea*.

For Kingdom Bacteria, the use of composite DNA signatures combining nDNA and pDNA (when available) resulted in separation in all cases.

Overall, the use of composite DNA signatures resulted in separation in all pairwise comparisons in Fig. 2 (where organellar DNA sequencing data was available), including in those where the use of conventional nDNA signature failed or resulted in inconclusive separations.

### Assembled DNA signatures

As the third objective of this study, we explored a way to enhance the practical applicability of conventional DNA signatures. Recall that, to produce a recognizable visual pattern that can be reliably used to represent a genome, a conventional DNA signature needs as input a long contiguous (two to several hundred kilobase pairs) DNA fragment. This assumes a high quality and reliability of sequencing and assembly, which are not always available. We propose instead to approximate a conventional signature by an assembled DNA signature, which combines the conventional DNA signatures of many short contigs (e.g., 100 bp) of the given fragment. Note that these contigs need not cover the entire DNA fragment.

In what follows, we denote by |*s*| the length of the sequence *s*. Given a DNA fragment *s*, an assembled DNA signature of *s*, using *r* equi-length contigs of length *n* (subfragments of the sequence *s*), is defined as the sum of the conventional DNA signatures of all of the *r* contigs. A particular case of assembled DNA signature is where the fragment *s* is partitioned into equi-length, consecutive, non-overlapping contigs, that is, *s*=*s*_1_*s*_2_…*s*_*r*_*s*_*r*+1_, and |*s*_*i*_|=*n* for 1≤*i*≤*r*, with |*s*_*r*+1_|<*n*. In this case, we call the assembled signature a fully-assembled DNA signature of the sequence *s*, using equi-length contigs of length *n*.

Table 1 ((A) through (C)) presents a comparison between the conventional nDNA signature of a given DNA fragment and its assembled DNA signatures, as well as fully-assembled DNA signatures, for various values of contig length *n*, and number of contigs *r*. The DNA fragment used is from *H. sapiens*, chromosome 21, fragment 20 (from position 2,850,001 to 3,000,000 after removing all *N*s in the original sequence), and the distance used is approximated information distance between CGRs. For example, the distance between the conventional nDNA signature and the fully-assembled DNA signature of the same fragment, that uses 1,000 contigs of length 150 bp each, is 0.03 (row 2, column (A)). This value is very small, given that approximated information distance theoretically ranges between 0 and 1. This suggests that, for these parameter values (*n*=150 and *r*=1,000), a fully-assembled DNA signature can be an excellent approximation of the conventional DNA signature of the same fragment. This was expected, given that the only information lost in the computation of a fully-assembled DNA signature, when using the approximated information distance, is the information about the *k*-mers situated at the borders between contigs.

**Table 1:** (*A*) through (*C*) – Distances between the conventional nDNA signature of a fragment and its assembled DNA signatures, for various numbers *r* of contigs of the same length *n*: (*A*) distances to fully-assembled DNA signatures; (*A* ^′^) theoretical upper bounds for (*A*); (*B*) distances to assembled DNA signatures; (*C*) same as (*B*), when tripling the number of contigs

*n*	*r*	(A)	(A’)	(B)	*r*	(C)	*r*	(B’)	*r*	(C’)
100	1500	0.05	0.13	0.29	4500	0.042	1475	0.32	4434	0.041
150	1000	0.03	0.09	0.29	3000	0.034	1000	0.29	2999	0.040
200	750	0.02	0.07	0.28	2250	0.033	750	0.29	2250	0.038
300	500	0.02	0.04	0.28	1500	0.030	500	0.28	1500	0.038
500	300	0.01	0.03	0.26	900	0.037	300	0.28	900	0.033
1000	150	0.005	0.01	0.30	450	0.030	150	0.25	450	0.039
2000	75	0.003	0.007	0.30	225	0.041	75	0.26	225	0.023
3000	50	0.002	0.004	0.25	150	0.044	50	0.29	150	0.021
10000	15	0.0004	0.001	0.30	45	0.053	15	0.25	45	0.045
15000	10	0.0003	0.0008	0.24	30	0.12	10	0.23	30	0.079
30000	5	0.0001	0.0004	0.36	15	0.13	5	0.41	15	0.058

(*B* ^′^)through (*C* ^′^) – Distances between the conventional nDNA signature of a fragment and its assembled DNA signatures, using variable-length contigs taken from a normal distribution *N*(*n*,*σ*), with mean *n* and variance *σ*=40. The nDNA fragment used was from *H. sapiens*, chromosome 21, fragment 20 (from position 2,850,001 to 3,000,000 after removing all *N*s in the original sequence)

Also as expected, for the same values of *n* and *r*, the distance between an assembled DNA signature and the conventional nDNA signature of the same fragment (Table 1, Column (B)) is higher than the one between a fully-assembled DNA signature and the conventional nDNA signature of the same fragment (Table 1, (A)). This indicates that the assembled DNA signature is less performant than the fully-assembled DNA signature as an approximation of a conventional nDNA signature. The reason is that, given a fixed number *r* of contigs, in the case of an assembled DNA signature the contigs are allowed to overlap and need not cover the entire fragment. This can be compensated by increasing the coverage, that is, the number *r* of contigs. Table 1, (C) shows that tripling the number of contigs results in significantly smaller differences between assembled DNA signatures and the conventional DNA signature of the same fragment which they were meant to approximate.

The results in Table 1 suggest that assembled DNA signatures have the potential to play the role of “genomic signatures”, and be used directly on raw unassembled next-generation sequencing read data, or in cases where other methods are not directly applicable because high-quality sequencing data is not available. To test this hypothesis, we considered the organism pairs in Fig. 2 for which separation was obtained using conventional nDNA signatures, and attempted to reproduce these successful separations using assembled DNA signatures instead. In addition, we empirically sought to find, in each case, the coverage (amount of sequence data) needed to achieve separation, as a percentage of total fragment length.

To determine the threshold interval where separation between assembled DNA signatures of a given pair of organisms was achieved, when contigs of length *n*=300 were used, the following process was employed. For various values of *t*, 0≤*t*≤1 (representing the fragment coverage, e.g., *t*=0.5 means that 50 % of the fragment data was used), we attempted to see if separation of assembled DNA signatures from the two organisms was achieved, in the following way.

For each of the 150 kbp fragments *s* from the two genomes, *q* random positive integers were picked from the interval 1 to |*s*|−*n*+1=(150,000−300+1), where *q*=⌊*t*∗|*s*|/*n*⌋, that is, the integer part of *t*∗|*s*|/*n*. These *q* numbers represent the start positions of the *q* chosen contigs. For each contig start position, a contig of length *n*=300 was read and used for the assembled DNA signature of the fragment *s*.

For each value of *t*, the corresponding 3D Molecular Distance Map of the assembled DNA signatures of the two organisms was then analyzed, by verifying the existence (or absence) of a separating plane.

The results are summarized in Table 2 and can be interpreted as follows. In the comparison between *H. sapiens* and *D. melanogaster* the threshold interval is 1 –5 %. The lower limit of this interval is 1 %, and this means that in the computation using the coverage value *t*=0.01 (implying *q*=⌊0.01∗150,000/300⌋=5), separation was not achieved. That is, for each of the 150 kbp nDNA fragments available (480 from *H. sapiens* and 128 from *D. melanogaster*), when employing assembled nDNA signatures using only 5 contigs per fragment (for a maximum of 1 % of each fragment length, that is, 1,500 bp per fragment), separation was not achieved. The upper limit of the interval is 5 %, and this means that in the computation using the coverage value *t*=0.05 (implying *q*=25), separation was achieved. That is, when employing assembled nDNA signatures using 25 contigs per fragment (for a maximum of 5 % of each fragment length, that is, 7,500 bp per fragment), separation was achieved.

**Table 2:** Assembled nDNA signatures: sequence coverage (amount of DNA fragment information) needed for separation of the assembled nDNA signatures of the pivot organism from assembled nDNA signatures of the comparison organism, for all major kingdoms of life. Separations were confirmed by verifying the existence of separating planes

**Animalia**		
*H*.*s* *a* *p* *i* *e* *n* *s*vs.	Different taxon	Thresh.
*D*.*m* *e* *l* *a* *n* *o* *g* *a* *s* *t* *e* *r*	Phylum: Arthropoda	1 –5 %
*G*.*g* *a* *l* *l* *u* *s*	Class: Aves	3 –10 %
*M*.*m* *u* *s* *c* *u* *l* *u* *s*	Order: Rodentia	10 –20 %
*M*.*m* *u* *r* *i* *n* *u* *s*	Suborder: Strepsirrhini	60 –80 %
*T*.*s* *y* *r* *i* *c* *h* *t* *a*	Infraorder: Tarsiiformes	20 –40 %
**Fungi**		
*S*.*c* *e* *r* *e* *v* *i* *s* *i* *a* *e* vs.	Different taxon	Thresh.
*C*.*g* *a* *t* *t* *i* *i*	Phylum: Basidiomycota	0.5 –2 %
*F*.*o* *x* *y* *s* *p* *o* *r* *u* *m*	Class: Sordariomycetes	0.5 –2 %
*K*.*p* *a* *s* *t* *o* *r* *i* *s*	Family: *Phaffomycetaceae*	2 –10 %
*C*.*d* *u* *b* *l* *i* *n* *i* *e* *n* *s* *i* *s*	Genus: *C* *a* *n* *d* *i* *d* *a*	2 –10 %
**Plantae**		
*B*.*n* *a* *p* *u* *s*vs.	Different taxon	Thresh.
*M*.*p* *u* *s* *i* *l* *l* *a*	Phylum: Chlorophyta	2 –3 %
*P*.*p* *a* *t* *e* *n* *s*	Unranked: Bryophyta	3 –4 %
*M*.*d* *o* *m* *e* *s* *t* *i* *c* *a*	Unranked: Fabids	4 –5 %
*C*.*p* *a* *p* *a* *y* *a*	Family: *Caricaceae*	4 –5 %
**Protista**		
*P*.*f* *a* *l* *c* *i* *p* *a* *r* *u* *m* vs.	Different taxon	Thresh.
*O*.*t* *r* *i* *f* *a* *l* *l* *a* *x*	Phylum: Ciliophora	0.5 –2 %
*T*.*g* *o* *n* *d* *i* *i*	Class: Conoidasida	0.5 –2 %
*T*.*o* *r* *i* *e* *n* *t* *a* *l* *i* *s*	Order: Piroplasmida	0.5 –2 %
*P*.*v* *i* *v* *a* *x*	Species: *P*.*v* *i* *v* *a* *x*	0.5 –2 %
**Bacteria**		
*E*.*c* *o* *l* *i* vs.	Different taxon	Thresh.
*S*.*a* *u* *r* *e* *u* *s*	Phylum: Firmicutes	0.5 –2 %
*H*.*p* *y* *l* *o* *r* *i*	Class: Epsilonproteobact.	0.5 –2 %
*A*.*b* *a* *u* *m* *a* *n* *n* *i* *i*	Order: Pseudomonadales	0.5 –2 %
*S*.*e* *n* *t* *e* *r* *i* *c* *a*	Genus: *Salmonella*	10 –20 %
**Archaea**		
*P*.*f* *u* *r* *i* *o* *s* *u* *s* vs.	Different taxon	Thresh.
*S*.*i* *s* *l* *a* *n* *d* *i* *c* *u* *s*	Phylum: Crenarchaeota	0.5 –2 %
*M*.*s* *m* *i* *t* *h* *i* *i*	Class: Methanobacteria	0.5 –2 %
*Thermococcus*	Genus: *Thermococcus*	0.5 –2 %
*P*.*y* *a* *y* *a* *n* *o* *s* *i* *i*	Species: *P*.*y* *a* *y* *a* *n* *o* *s* *i* *i*	0.5 –2 %

The actual threshold values lie in the intervals listed, and may be subject to the quality of the sequencing. As expected, in general, the thresholds needed for separation increase with the increase in the degree of relatedness of the organisms being compared. This suggests that nDNA sequences from closely related organisms require a higher coverage (that is, a higher amount of information from each sequence) to be separated. The only exception to this trend, in this dataset, were the pairs *H. sapiens* with *M. murinus* (gray mouse lemur) requiring 60 –80 % sequence coverage, and *H. sapiens* and *T. syrichta* (Philippine tarsier) requiring 20 –40 % sequence coverage. Thus, the (human, lemur) pair required higher sequence coverage to achieve separation than the (human, tarsier) pair, even though the gray mouse lemur belongs to a different primate suborder (Haplorrhini) than the modern human, while the tarsier belongs to the same primate suborder as the modern human (Strepsirrhini), and thus one would expect that more information would be needed to achieve the latter separation. This apparent anomaly may be partly related to the fact that the phylogenetic placement of tarsiers within the order Primates has been controversial for over a century [59]: In [60] tarsiers are placed within Haplorrhini, while according to [20, 61], mitochondrial DNA evidence places tarsiiformes as a sister group to Strepsirrhini.

Table 2 indicates that the amount of DNA fragment information needed to achieve separation, at the same taxonomic level, can differ from one kingdom to another. For example, in Kingdom Animalia, conventional nDNA signatures of organisms from two species of a different species (*H. sapiens* and *P. troglodytes*) could not be separated even though we use 100 % of the DNA fragment information. In contrast, in Kingdom Fungi, assembled nDNA signatures from two organisms of a different genus (*S cerevisiae* and *C. dubliniensis*) could be separated even when using only 10 % of DNA fragment data. Similarly, in Kingdom Bacteria, assembled nDNA signatures from two organisms of different genus (*E. coli* and *S. enterica*) could be separated even when using only 20 % of DNA fragment data. The situation is even more extreme in Kingdom Protista and Kingdom Archaea, where even organisms belonging to the same genus could be separated with very little sequence coverage. Indeed, in Kingdom Protista, assembled nDNA signatures of two organisms of the same genus (*P. falciparum* and *P. vivax*) could be separated using only 2 % of DNA fragment data. Similarly, in Kingdom Archaea, assembled nDNA signatures from two organisms of the same genus (*P. furiosus* and *P. yananosii*) could also be separated using only 2 % of DNA fragment data. This suggests that some taxonomic categories, such as “genus”, do not necessarily reflect the same degree of structural similarity of genomic sequences uniformly across kingdoms.

### Composite-assembled DNA signatures

We now briefly explore the potential of combining the approach of composite DNA signatures with that of assembled DNA signatures. A composite-assembled DNA signature is produced by combining information from the assembled DNA signatures of two (or more) different types of DNA fragments. For example, a composite-assembled signature using nDNA and mtDNA is obtained by combining the assembled nDNA signature of one 150 kbp nDNA fragment, with the assembled mtDNA signature of the mtDNA genome of the same organism.

**Fig. 6:**
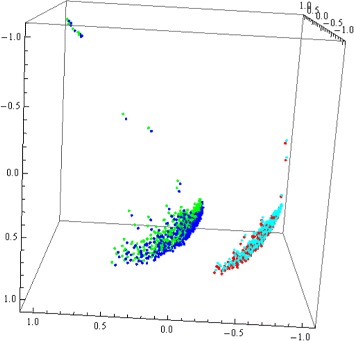
3D Molecular Distance Map illustrating interrelationships among 480 composite (respectively 480 composite-assembled) DNA signatures, each using one nDNA fragment and the mtDNA genome from *H. sapiens*, *blue* (resp. *green*); and 500 composite (resp. 500 composite-assembled) DNA signatures, each using one nDNA fragment and the mtDNA genome from *P. troglodytes*, *red* (resp. *turquoise*); For the composite-assembled DNA signatures, the length of contigs was *n*=100, while the number of contigs was 4,500 for each 150 kbp nDNA fragment, and 497 (resp. 496) for the human (resp. chimp) mtDNA genome. The accuracy of separation between the *H. sapiens* and the *P. troglodytes* sequences was 58 %, but the existence of a separation plane was verified

Figure 6 plots together composite DNA signatures and composite-assembled DNA signatures using nDNA and mtDNA from *H. sapiens* and *P. troglodytes*. Note that composite-assembled DNA signatures and composite DNA signatures of fragments (using nDNA and mtDNA), from the same species are closely clustered together. On the other hand, all DNA signatures of *H. sapiens* are separated from all DNA signatures of *P. troglodytes*, and the existence of a separating plane was verified. These results suggest that composite-assembled DNA signatures could also be potential candidates for the role of “genomic signature”, as they have in general better discriminating power than conventional nDNA signatures while using scattered and potentially less sequence information.

## Conclusions

The first objective of this paper was to conduct a comprehensive analysis, on a dataset totalling 1.45 Gb, of the hypothesis that Chaos Game Representations of nuclear/nucleoid genomic sequences can play the role of “genomic signatures”, that is, that they are genome- and species-specific. Our results suggest that this hypothesis is not always valid, in that nuclear/nucleoid DNA sequences belonging to closely related species such as *H. sapiens* and *P. troglodytes* or *E. coli* and *E. fergusonii* cannot always be separated using conventionally computed CGR signatures.

To address this issue, as a second objective, we propose the use of composite DNA signatures, which combine information from the nuclear/nucleoid genome with that from one or more organellar genomes (mtDNA, cpDNA and/or pDNA). Composite DNA signatures were found, in this study, to result in successful separation of DNA sequences by organism in all cases, including those where conventional nDNA signatures failed.

As a third objective, we propose the use of assembled DNA signatures, which combine information from short contigs (subfragments) of a DNA fragment, rather than using the entire contiguous fragment, to produce its signature. We show that assembled DNA signatures can be successful replacements of conventional DNA signatures, and also that the composite and assembled DNA signature approaches can be used simultaneously.

Mathematically, composite and assembled DNA signatures are both particular cases of a general concept, namely that of an additive DNA signature of a set of DNA sequences (see Section “Methods”). Our results indicate that such additive DNA signatures could be considered as potential candidates for the role of “genomic signatures” at various taxonomic levels, from distant to closely related species, and thus complement other methods for species identification and classification.

Several directions of future research stem from the fact that existing literature indicates that the oligomer composition of nuclear/nucleoid DNA sequences and mitochondrial DNA sequences can be a source of taxonomic information. Such directions include testing the discriminating power of additive DNA signatures in large-scale multi-genome comparisons, and exploring their utility in practical applications such as DNA sequence identification and classification (including directly on raw unassembled NGS read data or when high-quality sequencing data is not available), metagenomics, and synthetic genomes.

## Methods

### Dataset

The dataset, totalling 1.45 Gb, comprised whole nuclear/nucleoid genomes and organellar genomes of 42 organisms, spanning all major kingdoms of life (see Additional file 1 for the scientific name, NCBI accession number, chromosome number, and number of fragments sampled). In our analysis, for each complete genomic sequence, all letters other than *A, C*,*G, T* were ignored, and the resulting DNA sequence was divided into successive, non-overlapping, contiguous fragments, each 150 kbp long (when the last portion was shorter than 150 kbp, it was not included in the analysis). The choice of fragment length, 150 kbp, was due to our choice of CGR image resolution (namely 2^9^×2^9^, that is, *k*=9), empirical testing, and computational efficiency reasons, see [25].

Subsequently, 20 such 150 kbp fragments were randomly sampled from each chromosome and, for each such fragment, a corresponding conventional nDNA signature was constructed, as described below. (If there were fewer than 20 fragments, all fragments in the chromosome were chosen.) In the cases where the genome assembly of the organism was at the contig/scaffold level, the contigs/supercontigs of the assembly were sorted by length and the first 500 contigs/supercontigs were selected. (If there were fewer than 500 contigs/supercontigs, all were selected.) From each contig/supercontig, only the first 150 kbp fragment was considered.

We note that this method is alignment-free, and that its approach contrasts typical biodiversity and species identification research [62–65] in that it uses randomly selected DNA sequences rather than specific marker genes for identification and classification of species. This approach is somewhat similar to novel approaches in metagenomics, metatranscriptomics, and viromics [66], but there are also substantial differences such as that metatranscriptomics is based on RNA rather than DNA and that it groups sequences based on functionality rather than oligomer composition.

### Chaos Game Representation (CGR)

**Fig. 7:**
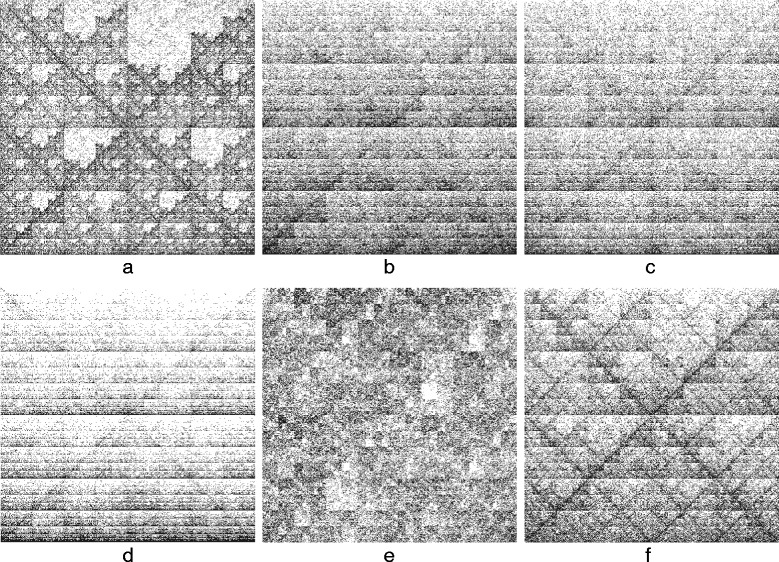
Conventional nDNA signatures of 150 kbp sequences of the pivot organisms from Kingdom **a** Animalia, **b** Fungi, **c** Plantae, **d** Protista, **e** Bacteria, and **f** Archaea

CGR is a method introduced by Jeffrey [1] as a way to visualize the structural composition of a DNA sequence. This method associates an image to each DNA sequence as follows: Starting from a square with corners labelled *A, C, G,* and *T*, and the center of the square as the starting point, the image is obtained by successively plotting each nucleotide as the middle point between the current point and the corner labelled by the nucleotide to be plotted. If the generated square image has a size of 2^*k*^×2^*k*^ pixels, then every pixel represents a distinct *k*-mer: A pixel is black if the *k*-mer it represents occurs in the DNA sequence, otherwise it is white. CGR images of genetic DNA sequences originating from various species show patterns such as squares, parallel lines, rectangles, triangles, and also complex fractal patterns, as shown in Fig. 7.

We used a modification of the original CGR, introduced by Deschavanne [3]: a *k*-th order FCGR (frequency CGR) of a sequence *s*, denoted by *FCGR*_*k*_(*s*), is a 2^*k*^×2^*k*^ matrix that can be constructed by dividing the CGR image of the sequence *s* into a 2^*k*^×2^*k*^ grid, and defining the element *a*_*ij*_ of the matrix *FCGR*_*k*_(*s*) as the number of points that are situated in the corresponding grid square.

We now formally define the conventional DNA signature of a sequence *s* to be the matrix *FCGR*_*k*_(*s*), which records the numbers of occurrences of all possible *k*-mers in the sequence *s*. Throughout this paper, the parameter *k* is assumed to be a fixed constant. In particular, similar to [25], in all computational experiments in this paper the value used was *k*=9.

For computing composite and assembled DNA signatures, we introduce the general concept of additive DNA signature of a set of sequences, formally defined as follows.

#### **Definition 1**

The additive DNA signature of a set of sequences *S*={*s*_1_,*s*_2_,…,*s*_*r*_},*r*≥1, is defined as

$${FCGR}_{k}(S) = {FCGR}_{k}(s_{1})+ \ldots + {FCGR}_{k}(s_{r}). $$

Note that the notions of conventional DNA signature, composite DNA signature, assembled DNA signature, and fully-assembled DNA signature, are all particular cases of additive DNA signatures, as follows:

- The conventional DNA signature of a sequence *s* is the additive DNA signature of the set {*s*} consisting of a single sequence *s*, that is, *FCGR*_*k*_(*s*)=*FCGR*_*k*_({*s*}).
- The composite DNA signature using two DNA sequences *s*_1_,*s*_2_, of two different types, is
  *FCGR*_*k*_({*s*_1_,*s*_2_})=*FCGR*_*k*_(*s*_1_)+*FCGR*_*k*_(*s*_2_),
- An assembled signature of a sequence *s*, using *r* equi-length contigs of length *n*, is
  ${FCGR}_{k}(\{s_{1}, s_{2}, \ldots, s_{r}\}) = \sum _{i = 1}^{r} {FCGR}_{k}(s_{i}),$ where *s*=*α*_*i*_*s*_*i*_*β*_*i*_,|*s*_*i*_|=*n*, for 1≤*i*≤*r*.
- The fully-assembled DNA signature of a sequence *s*, using equi-length contigs of length *n*, is
  ${FCGR}_{k}(\{s_{1}, s_{2}, \ldots, s_{r}\}) = \sum _{i=1}^{r} {FCGR}_{k}(s_{i}),$ where *r*=⌊|*s*|/*n*⌋,*s*=*s*_1_*s*_2_…*s*_*r*_*s*_*r*+1_, and |*s*_*i*_|=*n* for 1≤*i*≤*r*, while |*s*_*r*+1_|<*n*.

To compute the fully-assembled DNA signature of a sequence *s*, using equi-length contigs of length *n*, one adds the *FCGR*_*k*_ of all the adjacent consecutive contigs of length *n* that cover *s* (except possibly a short tail of length less than *n*), where the first contig starts at the beginning of the sequence. In contrast, to compute an assembled signature of *s* using equi-length contigs of length *n*, one has the freedom to set the number of such contigs as an additional parameter *r*, and then add the *FCGR*_*k*_ of *r* contigs sampled randomly from the sequence *s*. Thus, for a given *n*, a sequence *s* has only one fully-assembled DNA signature, but many different assembled signatures, each depending on both the choice of parameter *r*, and the particular sampling of the *r* sequences (which may overlap or be identical).

For example, if *s* is the DNA sequence

$$ s=AAAAACCCCCGGGGGTTT, $$

of length 18, and if we consider contigs of length *n*=5, then the fully-assembled DNA signature of *s* is unique and is obtained by adding the *FCGR*_*k*_ of the following *r*=⌊18/5⌋=3 contigs

$$ \{AAAAA,CCCCC,GGGGG\} $$

that cover *s* (except the discarded remainder *TTT*).

For the same sequence *s* and contig length *n*=5, many diferent assembled DNA signatures can be computed. For example, an assembled DNA signature of *s* using *r*=3 equi-length contigs of length *n*=5 could use contigs {*AAACC, CCCGG, CCCGG*}, while another could use contigs {*AACCC, CCCCG, GGTTT*}. In addition, other assembled DNA signatures of *s* with equi-length contigs of length *n*=5 exist, depending on the parameter *r*. For example, an assembled DNA signature of *s* using *r*=5 equi-length contigs of length *n*=5 could use the contigs

$$ \{AAAAA, AAACC, CGGGG, GGGGT, GGTTT \}. $$

### Approximated Information Distance (AID)

For a finite set *X*, we denote by |*X*| the cardinality of *X*, that is the number of elements in *X*. Given a set of sequences *S*={*s*_1_,*s*_2_,…,*s*_*n*_} we denote by *M*_*k*_(*S*) the set of all distinct *k*-mers that occur in all the sequences of *S*. In the case of a set consisting of a single sequence *s*, we write *M*_*k*_(*s*) to denote *M*_*k*_({*s*}).

The approximated information distance between two sequences *s* and *t* (introduced in [25] as a slight modification of a distance used in [53]) is defined as:

$$d_{\mathtt{AID}}^{k}(s,t) = \frac{|M_{k}(s)\setminus M_{k}(t)| + |M_{k}(t)\setminus M_{k}(s)|}{|M_{k}(\{s, t\})|}, $$

where for two sets *X* and *Y*, *X*∖*Y* denotes the set difference between *X* and *Y*, that is, the set of elements that belong to *X* but not to *Y*.

The distance *d*AID*k*(*s, t*) was used for most of the computations of pairwise distances between conventional DNA signatures in this paper.

The notion of approximated information distance between two sequences can now be extended to that of generalized approximated information distance between two sets of sequences *S* and *T*, as:

$$ d_{\mathtt{AID}}^{k}(S,T) = \frac{|M_{k}(S)\setminus M_{k}(T)| + |M_{k}(T)\setminus M_{k}(S)|}{|M_{k}(S\cup T)|}. $$

This generalization of the approximated information distance preserves the original meaning of the concept as the ratio between the number of noncommon *k*-mers of the two sets *S* and *T* and the total number of *k*-mers that occur in *S* or in *T* (or both). This distance was used to compute distances between conventional, composite and assembled DNA signatures in this paper.

The next Proposition leads to a formula for the computation of the generalized approximated information distance, as well as gives a theoretical upper bound for the generalized approximated information distance in the case of fully-assembled DNA signatures. The following auxiliary lemma follows from standard set theory arguments.

#### **Lemma 2**

Let *s* be a sequence and *S, T* be two finite sets of sequences over the DNA alphabet {*A, C*,*G, T*}, and let *k*≥2 be an integer. The following statements hold true.

1. If *S*⊆*T* then |*M*_*k*_(*S*)|≤|*M*_*k*_(*T*)| and
  |*M*_*k*_(*S*∪*T*)|=|*M*_*k*_(*T*)|,
2. If every sequence in *S* is a subsequence of a given sequence *s*, then
  |*M*_*k*_(*S*)∪*M*_*k*_(*s*)|=|*M*_*k*_(*s*)|,
3. The number of distinct *k*-mers that occur in *S* but not in *T* is |*M*_*k*_(*S*)∖*M*_*k*_(*T*)|=|*M*_*k*_(*S*∪*T*)|−|*M*_*k*_(*T*)|,
4. |*M*_*k*_(*S*)|=*#FCGR*_*k*_(*S*),

where for a numerical matrix *A* we denote by *#*(*A*) or *#A* the number of non-zero entries of *A*.

#### **Proposition 3**

Let *s* be a sequence and let *S, T* be two sets of sequences. The following statements hold true.

1. $d_{\mathtt {AID}}^{k}(S,T)= 2 - \frac {|M_{k}(S)|+|M_{k}(T)|}{|M_{k}(S \cup T)|}$
2. If *s*=*s*_1_*s*_2_…*s*_*r*_ and each *s*_*i*_ is of length *n*, *n*>*k*,
  then
  $d_{\mathtt {AID}}^{k}(\{s_{1}s_{2}\ldots s_{r}\}, s)\le \frac {\text {min}\{(r-1)(k-1), |M_{k}(s)|\}}{|M_{k}(s)|}.$
3. There is a sequence *s* for which the above relation holds with “=”.

#### *Proof*

The first statement follows from Lemma 2.3, by noting that *d*AID*k*(*S, T*) equals

$$\begin{aligned} \frac{\Big{(}|M_{k}(S \cup T)| - |M_{k}(T)| \Big{)} +\Big{(}|M_{k}(S \cup T)| - |M_{k}(S)| \Big{)}}{|M_{k}(S\cup T)|} \end{aligned} $$

which is indeed equal to the required formula.

For the second statement, let *S*={*s*_1_,*s*_2_,…,*s*_*r*_} and *T*={*s*}. By the definition of the generalized information distance, *d*AID*k*({*s*_1_,…,*s*_*r*_},*s*) equals a fraction, where the numerator is the sum between the number of distinct *k*-mers that appear in {*s*_1_,…,*s*_*r*_} but not in *s*, and the number of distinct *k*-mers that appear in *s* but not in {*s*_1_,…,*s*_*r*_}. The first term of this sum is obviously zero, since *s*_*i*_ are contigs that span the sequence *s*. Thus, the numerator of this fraction is the second term of the sum, namely the number of distinct *k*-mers that appear in *s*=*s*_1_*s*_2_…*s*_*r*_ but not in {*s*_1_,…,*s*_*r*_}. We can count these *k*-mers by noticing that the only *k*-mers that appear in *s* but not in {*s*_1_,…,*s*_*r*_}, are the ones that span consecutive contigs.

We now note that each joint of two contigs *s*_*i*_*s*_*i*+1_ contains at most (*k*−1) distinct *k*-mers that span both contigs, and that *s* contains (*r*−1) such joints *s*_*i*_*s*_*i*+1_. Thus, the total number of *k*-mers of *s*, that are in *s* but not in {*s*_1_,…,*s*_*r*_}, is at most (*r*−1)·(*k*−1).

Since the denominator of the fraction is, by Lemma 2.2, |*M*_*k*_(*s*)∪*M*_*k*_({*s*_1_,*s*_2_,…,*s*_*r*_})|=|*M*_*k*_(*s*)|, we have that

$$ d_{\mathtt{AID}}^{k}(\{s_{1},\ldots, s_{r}\},s)\le \frac{0 + (r-1)(k-1)}{|M_{k}(s)|}. $$

Since the approximated information distance ranges between 0 and 1, the required inequality follows.

For the third statement, an example of a sequence where the upper bound of the distance between the conventional DNA signature of the sequence and the fully-assembled DNA signature of its contigs is reached is the sequence

$$ s=AAAACCCCGGGGTTTT, $$

with *k*=3 and *n*=*r*=4. Then *s* contains exactly 10 different 3-mers, that is, |*M*_3_(*s*)|=10, and (*r*−1)·(*k*−1)/|*M*_3_(*s*)|=0.6. On the other hand, let *s*_1_=*AAAA, s*_2_=*CCCC, s*_3_=*GGGG, s*_4_=*TTTT*. Then we have |*M*_3_({*s*_1_,*s*_2_,*s*_3_,*s*_4_})|=4, since only 4 distinct 3-mers, namely AAA, CCC, GGG and TTT can be found in this set, and thus

$$d_{\mathtt{AID}}^{3}(\{s_{1},s_{2},s_{3},s_{4}\},s)=2-\frac{4+10}{10}=0.6, $$

which equals the given upper bound. □

Remark that, by Proposition 3.1, the generalized approximated distance between two sets of sequences *S* and *T* can be now computed as

$$ d_{AID}^{k} (S, T) = 2 - \frac{\#{FCGR}_{k}(S) + \# {FCGR}_{k}(T)}{\#({FCGR}_{k}(S) + {FCGR}_{k}(T))}, $$

which is the formula that was used for all generalized approximated information distance calculations in this paper.

Remark also that the upper bound determined in Proposition 3.2 for the generalized approximated information distance, in the case of the comparison between the conventional DNA signature of a sequence and the fully-assembled DNA signature of its *r* contigs of length *n*, is the one illustrated in Column (*A*^′^) of Table 1.

### Multi-dimensional scaling and separation assessment

To visualize the interrelationships among DNA signatures originating from a pair of genomes, and thus to visually assess whether separation was achieved, we used Multi-Dimensional Scaling (MDS). MDS is an information visualization technique introduced by Kruskal in [67]. MDS takes as input a distance matrix that contains the pairwise distances among a set of items (here the items are DNA signatures), and outputs a spatial representation of the items in a common Euclidean space. Each item is represented as a point, and the spatial distance between any two points corresponds to the distance between the items in the distance matrix. Objects with a smaller pairwise distance will result in points that are close to each other, while objects with a larger pairwise distance will become points that are far apart.

Concretely, classical MDS, which we use in this paper, receives as input an *m*×*m* distance matrix (*Δ*(*i, j*))_1≤*i, j*≤*m*_ of the pairwise distances between any two items in the set. The output of classical MDS consists of *m* points in a *q*-dimensional space whose pairwise spatial (Euclidean) distances are a linear function of the distances between the corresponding items in the input distance matrix. More precisely, MDS will return *m* points $p_{1},p_{2},\ldots,p_{m}\in \mathbb {R}^{q}$ such that $d(i, j)= ||p_{i}-p_{j}||\thickapprox f(\Delta (i,j))$ for all *i, j*∈{1,…,*m*} where *d*(*i, j*) is the spatial distance between the points *p*_*i*_ and *p*_*j*_, and *f* is a function linear in *Δ*(*i, j*). Here, *q* can be at most (*m*−1) and the points are recovered from the eigenvalues and eigenvectors of the input *m*×*m* distance matrix. If we choose *q*=3, the result of classical MDS is an approximation of the original (*m*−1)-dimensional space as a three-dimensional map, such as the Molecular Distance Maps in this paper. Throughout the paper, for consistency, all Molecular Distance Maps have been scaled so that the *x*-, *y*-, and *z*- coordinates always span the interval [−1,1]. The formula used for scaling is $x_{\text {sca}} =2 \cdot \left (\frac {x - x_{\text {min}}}{x_{\text {max}} - x_{\text {min}}}\right) - 1$, where *x*_min_ and *x*_max_ are the minimum and maximum of the *x*-coordinates of all the points in the original map, and similarly for *y*_sca_ and *z*_sca_. In all Molecular Distance Maps displayed in this paper, the origin of coordinates (0,0,0) is the center of the depicted cube, and the parallel edges of the cube are parallel to one of the *x*-, *y*-, and *z*- axis respectively. The maps have been rotated for optimal visualization and, for each of the axes, the length units are displayed only on one of the four edges of the cube that are parallel to it.

A feature of MDS is that the points *p*_*i*_ are not unique. Indeed, one can translate or rotate a map without affecting the pairwise spatial distances *d*(*i, j*)=||*p*_*i*_−*p*_*j*_||. In addition, the obtained points in an MDS map may change coordinates when more data items are added to, or removed from, the dataset. This is because MDS aims to preserve only the pairwise spatial distances between points, and this can be achieved even when some of the points change their coordinates. In particular, the (*x, y*,*z*)-coordinates of a point representing the DNA signature of a particular DNA fragment of *H. Sapiens* in Fig. 1 will not be the same as the (*x, y*,*z*)-coordinates of the point representing the same DNA fragment in Fig. 3.

For a given Molecular Distance Map, *k-means clustering* [57] was used to assess whether separation of the DNA sequences by organism was achieved. The reason for this choice were that in all computed Molecular Distance Maps the number of clusters was known *a priori*, *k*=2 (not to be confused with *k*-mers, where *k* has a different meaning), that the clusters had approximately the same number of points and thus the prior probability of the two clusters was the same, and that in most cases the clusters were somewhat spherical in shape. Moreover, the use of *k*-means yielded satisfactory results in the majority of cases.

The *k*-means clustering algorithm proceeds as follows. Suppose *S*_1_ is the set of points originating from the genome of one of the organisms, and *S*_2_ is the set of points originating from the second one. *k*-means assigns labels *A* and *B* to all given points, in its attempt to cluster them into two clusters, *A* and *B*. The *k*-means accuracy score is computed by counting how many points were assigned correctly to their cluster, that is,

$$Acc = \frac{max \{ |A_{S_{1}}| + |B_{S_{2}}|, \;\; |B_{S_{1}}| + |A_{S_{2}}| \}}{|S_{1}| +|S_{2}|} $$

where $A_{S_{1}}$ is the set of points in the cluster *A* that belong to the set *S*_1_, and $B_{S_{2}}$ is the set of points in the cluster *B* that belong to the set *S*_2_ ($B_{S_{1}}$ and $A_{S_{2}}$ are defined similarly). If label *A* would correspond to species *S*_1_, and *B* to species *S*_2_, the quantity $ |A_{S_{1}}| + |B_{S_{2}}|$ would represent the number of points that have been correctly classified in this Molecular Distance Map, while $ |B_{S_{1}}| + |A_{S_{2}}|$ would represent the number of points that have been incorrectly classified. As a number, *Acc* is a quantity between 0.5 and 1, with 50 % indicating the worst clustering, and 100 % indicating perfect clustering. For this paper, any Molecular Distance Map with an accuracy greater than 85 % was interpreted as achieving separation of points by species.

In some cases the accuracy was less than 85 % in spite of the fact that separation of clusters could clearly be observed visually. A closer look at those cases revealed that they were generally plots similar to Fig. 4, that is, consisting of two long and thin clusters. In addition, in those plots the clusters were closer to each other than in Fig. 4. In such cases, *k*-means erroneously labelled the top halves of the two clusters by *A*, and the two bottom halves by *B*. For such situations, where the *k*-means clustering algorithm had a relatively low accuracy score but visual separation was nevertheless observed, we verified the existence of a plane that completely separated the two clusters. That is, if cluster *S*_1_ had *n*_1_ points of coordinates $(x_{i_{1}}, x_{i_{2}}, x_{i_{3}})$, where 1≤*i*≤*n*_1_, and cluster *S*_2_ had *n*_2_ points $(y_{j_{1}}, y_{j_{2}}, y_{j_{3}})$, where 1≤*j*≤*n*_2_, then our Mathematica-based code [68] was used to find one (out of possibly infinitely many) solutions to the system of equations with unknowns *a, b*,*c, d*:

$$\left\{ \begin{array}{lclr} a\cdot x_{i_{1}} + b\cdot x_{i_{2}} + c\cdot x_{i_{3}} + d & > 0, & i = 1,\ldots, n_{1}\\ a\cdot y_{j_{1}} + b\cdot y_{j_{2}} + c\cdot y_{j_{3}} + d & < 0, & j = 1,\ldots, n_{2} \end{array} \right. $$

that is, it found the equation *ax*+*by*+*cz*+*d*=0 of a plane with the property that the points of the cluster *S*_1_ are situated on one of its sides, while those of cluster *S*_2_ are situated on the other. For example, in Fig. 6, the equation of a plane computed by this method, that completely separates the points originating from *H. sapiens* from those originating from *P. troglodytes*, is *x*+0.918 *y*+0.37 *z*+0.0002=0.

For Molecular Distance Maps with more complex cluster shapes, where *k*-means accuracy is low and separating planes do not exist, the use of other clustering methods such as density-based spatial clustering of applications with noise (DBSCAN) [69] would have to be explored to see if separation is achieved.

The webtool MoDMap3D, [58], illustrates the 3D Molecular Distance Maps that correspond to each of the comparisons listed in Fig. 2, in the same way the Molecular Distance Map in Fig. 1 illustrates the positive separation result listed in Fig. 2, subfigure Animalia, line 1. The webtool MoDMap3D is, moreover, interactive, and allows for an in-depth exploration of each particular 3D Molecular Distance Map. After first selecting the pair of genomes to be compared, the user can navigate in the three-dimensional space of their DNA signatures: clicking on any point in the map will display information about the DNA fragment represented by that point, such as its NCBI accession number or assembly number, scientific name of the organism it originates from, chromosome or contig/scaffold number, length of the subsequence in bp, and fragment number from the original sequence.

### Software

The code for running the experiments [68] was written in Wolfram Mathematica, and was used for the generation of FCGRs, the computation of composite and assembled DNA signatures, the calculation of distance matrices, the creation of the 3D Molecular Distance Maps, and the computation of the separating planes.

### Remarks

One observation should be made about the genome assemblies at contig/scaffold level in the dataset. The general intent was for the 150 kbp DNA fragments from a given genome not to be overlapping. This is because sequence overlaps could result in artificially smaller intragenomic distances due to the increase in sequences’ similarities, and this could potentially lead to false positive cluster separations. However, some overlap may have been unavoidable in the cases where only contig/scaffold level data was available. The availability of contig/scaffold data only may thus explain why in Fig. 2 the accuracy scores do not always decrease uniformly, as expected, when one compares the pivot organism with organisms more and more closely related to it.

Another observation should be made about the length of sequences analyzed. When computing composite DNA signatures, the signature of the mitochondrial genome (or entire chloroplast or plasmid) was appended to that of each 150 kbp nDNA fragment. This, in some sense, magnifies the role of the organellar genome in the composite signature. Depending on the application, one can generalize Definition 1 to a weighted additive DNA signature which gives different weights to the different types of DNA that compose it.

We now discuss some limitations of the proposed methods. First, note that assembled DNA signatures as defined here use equi-length contigs. Preliminary computational experiments, illustrated in Table 1, columns (*B*^′^) and (*C*^′^), show the results of comparisons between a conventional nDNA signature and variable-length assembled DNA signatures of the same fragment. In those experiments, contig lengths are drawn from a normal distribution *N*(*μ*,*σ*) with mean *μ*=*n* (the length of the contig in the corresponding equi-length contig experiment) and variance *σ*=40. The table shows that the performance of assembled DNA signatures using variable-length contigs is comparable with the performance of those using equi-length contigs. This indicates that both equi-length and variable-length contigs assembled DNA signatures could be reliable approximations of conventional genomic signatures, depending on the application. Additional exploration is needed to confirm this hypothesis.

Second, every computational experiment in this study is a comparison between DNA signatures of genomic sequences belonging to two different organisms. Further analysis is needed to determine if the positive preliminary results on the discriminating power of composite and composite-assembled DNA signatures extend successfully to multi-genome comparisons. A necessary step for such an experiment would be a thorough investigation of intragenomic variations of FCGRs and finding a method to determine, for each genome, a single “representative” FCGR matrix to successfully represent that genome.

**Fig. 8:**
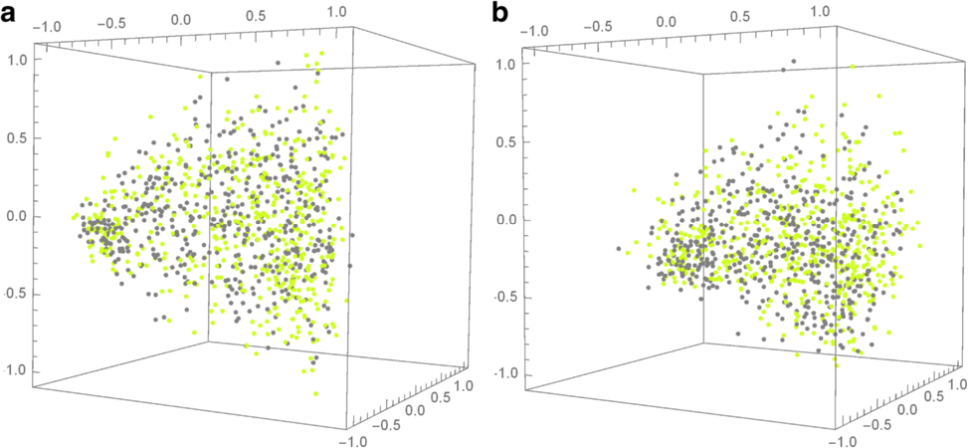
**a** Conventional nDNA signatures, and **b** composite (nDNA + cpDNA) signatures of *Capsicum annuum L*, cultivar *Zunla-1* (domesticated) shown in *light green*, and *Capsicum annuum* var. *glabriusculum*, cultivar *Chiltepin* (wild) shown in *grey*

Third, we mention a case where separation by organism could not be achieved, even when using composite DNA signatures (nDNA and cpDNA). This is the pairwise comparison between a cultivated pepper *Capsicum annuum L*, cultivar *Zunla-1* (domesticated) and its wild progenitor *Capsicum annuum* var. *glabriusculum*, cultivar *Chiltepin* (wild), see Fig. 8.

Several directions of future research stem from the observation that the function *FCGR*_*k*_ is a quasi-homomorphism from the set of all DNA sequences with the operation of catenation, to the set of 2^*k*^×2^*k*^ matrices with the operation of addition, in the sense that for sequences *s, t*, we have

$${FCGR}_{k}(st) \approx {FCGR}_{k}(s) + {FCGR}_{k}(t). $$

The definition of *FCGR*_*k*_ can be easily modified to make it an exact homomorphism by, e.g, defining a marked catenation of sequences *s* and *t* as *s*·*t*=*s$t*, with $ a new symbol, and constructing *FCGR*_*k*_ so as to not count any *k*-mer that includes the symbol $. Next steps in the exploration of the mathematical properties of additive DNA signatures include studying the implications of the homomorphic, structure-preserving, nature of *FCGR*_*k*_, as well as extensions of the concept of additive DNA signature, to, e.g., weighted additive DNA signatures which would give different weights to the different types of DNA that compose it.

## Abbreviations

AID, approximated information distance; CGR, chaos game representation; cpDNA, chloroplast DNA; FCGR, frequency CGR; MDS, multi dimensional scaling; mtDNA, mitochondrial DNA; nDNA, nuclear/nucleoid DNA; pDNA, plasmid DNA.

## Additional file

Additional file 1 The 42 genomes analyzed in the Results section, and the two genomes exemplified in the Remarks subsection: Scientific name, number of chromosomes, NCBI accession number. (∗*P.patens* genome from JGI Phytozome). (PDF 127 kb)
