# Supplementary material for: Additive methods for genomic signatures
Source: BMC Bioinformatics. 2016 Aug 22;17(1):313. doi: 10.1186/s12859-016-1157-8 (PMC4994249; doi:10.1186/s12859-016-1157-8)
Supplement: Additional file 1 — The 42 genomes analyzed in the Results section, and the two genomes exemplified in the Remarks subsection: Scientific name, number of chromosomes, NCBI accession number. (∗P.patens genome from JGI Phytozome). (PDF 127 kb) [file 12859_2016_1157_MOESM1_ESM.pdf]

Additional file 1

Additive Methods for Genomic Signatures

Rallis Karamichalis, Lila Kari, Stavros Konstantinidis,
Steffen Kopecki and Stephen Solis-Reyes

The 42 genomes analyzed in the Results section, and the two genomes exemplified in the Remarks subsection: Scientific name, number of chromosomes, NCBI accession number. (∗ *P.paterns* genome from JGI Phytozome).

Animalia

Organism	# Chrom.	# fragm.	Accession
Homo sapiens	24	480	GCF_000001405.30
Drosophila melanogaster	7	128	GCA_000001215.4
Gallus gallus	31	562	GCA_000002315.2
Mus musculus	21	420	GCA_000001635.5
Microcebus murinus	Contigs	500	ABDC02000001-ABDC02056281
Tarsius syrichta	Contigs	500	ABRT02000001-ABRT02492902
Callithrix jacchus	24	477	GCA_000004665.1
Papio anubis	21	420	GCF_000264685.2
Nomascus leucogenys	26	520	GCA_000146795.3
Pongo abelii	24	480	GCF_000001545.4
Gorilla gorilla gorilla	24	480	GCA_000151905.1
Pan troglodytes	25	500	GCA_000001515.4

Fungi

Organism	# Chrom.	# fragm.	Accession
Saccharomyces cerevisiae	16	73	GCF_000146045.2
Cryptococcus gattii	14	114	GCA_000185945.1
Fusarium oxysporum	15	265	GCA_000149955.2
Komagataella pastoris	4	58	GCA_000027005.1
Candida dubliniensis	8	93	GCA_000026945.1
Saccharomyces arboricola	16	66	GCA_000292725.1

Plantae

Organism	# Chrom.	# fragm.	Accession
Brassica Napus	19	380	GCF_000686985.1
Micromonas sp. RCC299	17	131	GCF_000090985.2
Physcomitrella patens	27	540	∗
Malus domestica	17	340	GCF_000148765.1
Carica papaya	Scaffold	231	DS981520-DS984726
Arabidopsis thaliana	5	100	GCF_000001735.3
Raphanus sativus	Scaffold	375	DF196826-DF236948
Brassica oleracea	9	180	GCF_000695525.1
Capsicum annuum L, cultivar Zunla-1	12	240	GCA_000710875.1
Capsicum annuum var. glabriusculum, cultivar Chiltepin	12	240	GCA_000950795.1

Protista

Organism	# Chrom.	# fragm.	Accession
Plasmodium falciparum	14	149	GCA_000002765.1
Oxytricha trifallax	Contigs	122	ARYC01000001-ARYC01025720
Toxoplasma gondii	Contigs	126	AAYL02000001-AAYL02002110
Theileria orientalis	4	58	GCA_000740895.1
Plasmodium vivax	14	142	GCA_000002415.2

Bacteria

Organism	# Chrom.	# fragm.	Accession
Escherichia coli	1	20	GCA_000183345.1
Staphylococcus aureus	1	18	GCA_000013425.1
Helicobacter pylori	1	11	GCA_000008525.1
Acinetobacter baumannii	1	20	GCA_000018445.1
Salmonella enterica	1	20	GCA_000006945.1
Escherichia fergusonii	1	20	GCA_000026225.1

Archaea

Organism	# Chrom.	# fragm.	Accession
Pyrococcus furiosus	1	12	GCA_000275605.1
Sulfolobus islandicus	1	18	GCA_000022385.1
Methanobrevibacter smithii	1	12	GCA_000016525.1
Thermococcus sp. AM4	1	13	GCA_000151205.2
Pyrococcus yayanosii	1	11	GCA_000215995.1
